# Cockleburr Removed without Instruments from Air Passage of Boy by Intra-Laryngeal Manipulations

**Published:** 1893-04

**Authors:** E. J. Sprathing

**Affiliations:** Opelika, Ala.


### COCKLEBURR REMOVED WITHOUT INSTRUMENTS FROM AIR PASSAGE OF BOY BY INTRALARYNGEAL MANIPULATIONS.

BY E J. SPRATHING, B. S., M. D., OPELIKA, ALA.

About 9 o'clock on the night of January 20th, 1892, I was sent for in great haste to go a short distance into the country. I found a boy twelve years old, who, while running through some weeds, had drawn into his air passage a cockleburr. His general appearance indicated speedy dissolution from asphyxia. Not even having had the nature of the trouble hinted to me until the bedside was reached, I found myself there with only a little pocket-case of medicines—not an instrument of any kind. The light was very poor, consisting of a lone candle. "'Tis the poor carpenter who grumbles at his tools." Mine were my long, bony fingers only. Under the exigences of the case it seemed extremely inappropriate to sit idly by while instruments for doing tracheotomy or laryngotomy were being brought from the city. My first thought was of emesis, which was at once dismissed as being impractical and cruel. Sneezing was tried with only an increase of the struggling for breath. Coughing was already excessive. Then the larnyx was firmly grasped by the thumb and fingers of the left hand, and forced upward with all might, while the middle finger of the right hand was insinuated beneath the epiglotis, between the false cords, when the burr was felt tightly wedged between the true vocal cords, but I could not move it from its lodging. At this juncture Dr. J. W. R. Williams came in with another nseless case of medicine. I briefly stated the trouble to him and asked him what was to be done. He said "Touch it again, and try a little harder to move it, I reckon." We had made a few more unsuccessful attempts when Dr. Williams suddenly exclaimed, "I believe I moved it." But was unable to lift it from its position. At this time it seemed that our patient would die from asphyxia, in spite of our anxiety, to say nothing of our futile efforts. I then forced—I say forced advisedly—the middle and ring fingers both into the larnyx at the same time, strained it upward with all might with the other hand, caught the burr between the two inserted fingers, turned it over, and drew it out, stem end foremost. Relief to the patient was instantaneous and complete. Saw him next day: said his throat was "sort of sore," also some swelling. Gave a tonic acid gargle. A week later he reported to me "as well as ever."

I think almost any movable foreign body can be removed from even the traches, by taking advantage of gravity in the position of the patient and manipulating in the proper manner.

